# Trained Innate Immunity Induced by Vaccination with Low-Virulence *Candida* Species Mediates Protection against Several Forms of Fungal Sepsis via Ly6G^+^ Gr-1^+^ Leukocytes

**DOI:** 10.1128/mBio.02548-21

**Published:** 2021-10-19

**Authors:** Elizabeth A. Lilly, Breah E. Bender, Shannon Esher Righi, Paul L. Fidel, Mairi C. Noverr

**Affiliations:** a Department of Microbiology and Immunology, Tulane University School of Medicine, New Orleans, Louisiana, USA; b Center of Excellence in Oral and Craniofacial Biology, Louisiana State University Health Sciences Center School of Dentistry, New Orleans, Louisiana, USA; University of Texas Health Science Center

**Keywords:** trained innate immunity, *Candida* species, myeloid-derived suppressor cells, candidemia, systemic *Candida* infections, *Candida*, immunization, myeloid-derived suppressor cell, sepsis

## Abstract

We recently discovered a novel form of trained innate immunity (TII) induced by low-virulence *Candida* species (i.e., Candida dubliniensis) that protects against lethal fungal/bacterial infection. Mice vaccinated by intraperitoneal (i.p.) inoculation are protected against lethal sepsis following Candida albicans*/*Staphylococcus aureus (*Ca*/*Sa*) intra-abdominal infection (IAI) or *Ca* bloodstream infection (BSI). The protection against IAI is mediated by long-lived Gr-1^+^ leukocytes as putative myeloid-derived suppressor cells (MDSCs) and not by prototypical trained macrophages. This study aimed to determine if a similar TII mechanism (Gr-1^+^ cell-mediated suppression of sepsis) is protective against BSI and whether this TII can also be induced following intravenous (i.v.) vaccination. For this, mice were vaccinated with low-virulence *Candida* strains (i.p. or i.v.), followed by lethal challenge (*Ca*/*Sa* i.p. or *Ca* i.v.) 14 days later, and observed for sepsis (hypothermia, sepsis scoring, and serum cytokines), organ fungal burden, and mortality. Similar parameters were monitored following depletion of macrophages or Gr-1^+^ leukocytes during lethal challenge. The results showed that mice vaccinated i.p. or i.v. were protected against lethal *Ca*/*Sa* IAI or *Ca* BSI. In all cases, protection was mediated by Ly6G^+^ Gr-1^+^ putative granulocytic MDSCs (G-MDSCs), with no role for macrophages, and correlated with reduced sepsis parameters. Protection also correlated with reduced fungal burden in spleen and brain but not liver or kidney. These results suggest that Ly6G^+^ G-MDSC-mediated TII is induced by either the i.p. and i.v. route of inoculation and protects against IAI or BSI forms of systemic candidiasis, with survival correlating with amelioration of sepsis and reduced organ-specific fungal burden.

## INTRODUCTION

Lethal sepsis is a common sequela of intra-abdominal infections (IAI) if left untreated or misdiagnosed (1, 2). Our laboratory has been studying fungal/bacterial sepsis using an experimental mouse model of Candida albicans/Staphylococcus aureus (*Ca*/*Sa*) polymicrobial IAI which results in 80 to 90% mortality by 48 to 72 h postinoculation (3–5). Characterization of host responses during *Ca*/*Sa* polymicrobial IAI demonstrated that mortality is associated with robust inflammation, evidenced by elevated levels of hallmark sepsis-associated proinflammatory mediators (interleukin 6 [IL-6], tumor necrosis factor alpha [TNF-α], IL-1β, and prostaglandin E_2_ [PGE_2_]) both locally and systemically, which can be abrogated by treatment with nonsteroidal anti-inflammatory drugs (NSAID) or by targeting PGE_2_-signaling pathways (4, 5).

Subsequent studies using non-*albicans Candida* (NAC) species in the primary challenge resulted in various levels of mortality. Of these, coinfection with Candida dubliniensis (*Cd;* a close phylogenetic relative of C. albicans) and S. aureus resulted in minimal mortality (∼10 to 20%). Interestingly, surviving mice were highly protected (80 to 90%) against a lethal intraperitoneal (i.p.) challenge with *Ca*/*Sa* 14 days later. Subsequent studies demonstrated that animals inoculated with a monomicrobial primary i.p. challenge (vaccination) with C. dubliniensis were equally highly protected (80 to 90%) against lethal *Ca*/*Sa* IAI. This protection was long-lived (up to 60 days post-*Cd* vaccination-challenge), but not mediated by adaptive immunity, with protection maintained in RAG1^−/−^ mice lacking T and B cells. Instead, protection appears to have been mediated by trained innate immunity (TII) that limits infection-associated sepsis. In this model, clodronate-mediated depletion of phagocytic macrophages failed to abrogate protection (6). Rather, a large influx of Gr-1^+^ (granulocyte receptor 1) leukocytes as early as 4 h post-lethal challenge in primary-challenged mice and the subsequent abrogation of protection following antibody depletion of Gr-1^+^ cells indicated a novel role for polymorphonuclear neutrophils (PMNs) in mediating protection. With the protective cells functional for at least 60 days postvaccination, and considering the short life span (24 h) of PMNs, these results suggested that the protective Gr-1^+^ cells were putative, long-lived myeloid-derived suppressor cells (MDSCs), which have been reported for other models of sepsis (7) and for patients with candidiasis (8).

Most recently, we further interrogated this novel form of TII by examining the microbial requirements and spectrum of the protective response. These studies revealed that in addition to *Cd*, several other low-virulence fungal species (Saccharomyces cerevisiae, Candida auris, Candida glabrata, and a C. albicans *efg1*Δ/Δ *cph1*Δ/Δ double-null mutant), irrespective of the ability to form true hyphae, conferred similar protection upon *Ca*/*Sa* lethal challenge (9). Additional characterization revealed the ability of these low-virulence *Candida* species to invade bone marrow by 24 h postvaccination, with a positive correlation between femoral bone marrow fungal infiltration and protection against the lethal IAI challenge. In contrast, while virulent C. albicans also infiltrates the bone marrow, there was more evidence of tissue/cellular damage concomitant with reduced protection against lethal challenge. Finally, it was revealed that this protection extended to a lethal intravenous (i.v.) *Ca* challenge, but with delayed mortality rather than long-term survival. This interesting observation prompted us to design experiments to understand the role of sepsis in the protected mice with delayed mortality, the role of sepsis in the intravenous model in general, and whether a similar Gr-1^+^ MDSC-mediated TII protection occurs in all the permutations of the systemic model: i.p. vaccination followed by i.v. *Ca* lethal challenge, as well as i.v. vaccination followed by *Ca* i.v. or *Ca/Sa* i.p. lethal challenge. There are two main subpopulations of Gr-1^+^ MDSCs, monocytic Ly6C^+^ leukocytes (M-MDSCs) and granulocytic Ly6G^+^ leukocytes (G-MDSCs). The relative contribution of each subset varies depending on the experimental model/disease, with adoptive transfer of G-MDSCs reported to ameliorate sepsis (10–12). Therefore, we also evaluated the contribution of G-MDSCs in mediating trained innate immunity in each model of systemic candidiasis.

## RESULTS

### Relative role of leukocyte populations in mediating trained innate immune protection induced by i.p. vaccination followed by i.v. lethal challenge.

**FIG 1:**
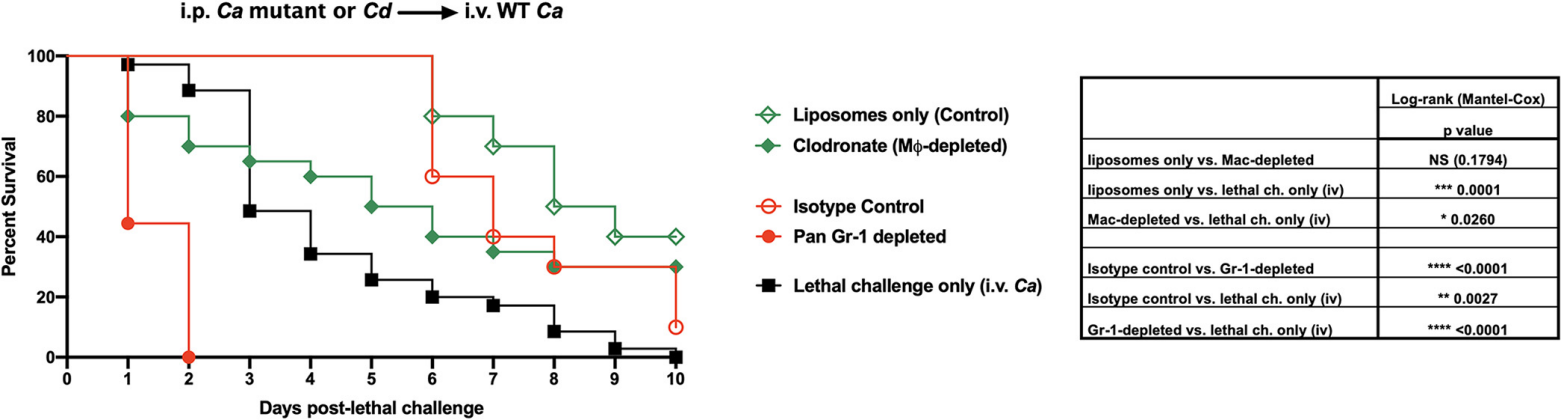
Relative role of leukocyte populations in mediating trained innate immune protection induced by intraperitoneal vaccination followed by intravenous challenge. Mice were given intraperitoneal (i.p.) primary challenge (vaccination) of the C. albicans *efg1*Δ/Δ *cph1*Δ/Δ mutant or C. dubliniensis 14 days prior to intravenous (i.v.) (C. albicans) lethal challenge. Mice (*n* = 10/group) given the primary challenge (i.p.) 14 days prior were injected i.p. with liposome-encapsulated clodronate (which results in ∼90% depletion of resident peritoneal macrophages) 1 day prior to lethal challenge or 200 μg anti-Gr-1 (Ly6G/C) antibody to deplete Gr-1^+^ leukocytes 48 h prior to and 2 h after lethal challenge. Antibodies were given every 2 days to the remaining live animals for the duration of the study. Liposomes alone and isotype control antibodies were included as controls. For all studies, mice were monitored for 10 days post-lethal challenge. Animals receiving no primary challenge served as the positive (lethal) controls. Results shown are cumulative of 8 independent experiments. Data were analyzed using the log-rank (Mantel-Cox) test. Actual *P* values are listed in the table. ****, *P* < 0.0001; ***, *P* < 0.001; **, *P* < 0.01; *, *P* < 0.05. NS, not significant.

Based on previous results showing a protective role for Gr-1^+^ leukocytes induced by intraperitoneal (i.p.) vaccination with avirulent *Candida* species against i.p. lethal *Ca*/*Sa* challenge, together with protection against i.v. lethal *Ca* challenge (9), we sought to determine the cell population(s) responsible for the protection against i.v. challenge. We also confirmed that there is no difference in the abilities of our avirulent strains (*Cd* or C. albicans *efg1*Δ/Δ *cph1*Δ/Δ double-null mutant) to induce protection; therefore, both were used throughout, with results combined. For this, mice (*n* = 10/group) administered the primary avirulent *Candida* strain vaccination (*Cd* or C. albicans *efg1*Δ/Δ *cph1*Δ/Δ double-null mutant i.p.) 14 days prior were injected i.p. with liposome-encapsulated clodronate or liposomes only (vehicle control) 1 day prior to i.v. lethal challenge or with anti-Gr-1 (Ly6G/C) antibodies or isotype control antibodies 48 h prior to, 2 h after lethal i.v. challenge, and every 2 days thereafter to the remaining live animals until the endpoint at 10 days. Results show no significant differences in survival between animals treated with liposomal clodronate and liposome vehicle alone, suggesting no role for macrophages in this protection (Fig. 1). In contrast, vaccinated mice given anti-Gr-1 antibodies, but not isotype control antibodies, followed by i.v. lethal challenge all succumbed by day 2 (Fig. 1). Efficacy of cellular depletion methods was confirmed by flow cytometry for clodronate-depleted F4/80^+^ macrophages (see Fig. S1 in the supplemental material) and anti-Ly6G/C-depleted Gr-1^+^ leukocytes (Fig. S2).

10.1128/mBio.02548-21.1 FIG S1 Efficacy of clodronate administration in depletion of F4/80^+^ macrophages. Mice (*n* = 3/group) were administered liposomal clodronate or liposomes alone intraperitoneally (i.p.). After 24 h, mice were sacrificed and spleens were collected. Splenocytes were incubated with fluorophore-conjugated anti-F4/80 antibody and analyzed by flow cytometry to confirm depletion of F4/80^+^ cells (macrophages). Numbers shown in the gray areas (A) and represented in the graph (B) are the percentage of F4/80^+^ cells in the spleens of naive (control) mice compared to mice treated with liposomes alone and liposomal clodronate. Data were analyzed using the Student’s *t* test. **, *P* < 0.01. Download FIG S1, TIF file, 1.8 MB. Copyright © 2021 Lilly et al. 2021 Lilly et al. https://creativecommons.org/licenses/by/4.0/ This content is distributed under the terms of the Creative Commons Attribution 4.0 International license.

10.1128/mBio.02548-21.2 FIG S2 **Efficacy of Gr-1 antibody administration in depletion of Gr-1^+^ leukocyte populations.** Mice (*n* = 5/group) were inoculated i.p. or intravenously (i.v.) with the C. albicans *efg1*Δ/Δ *cph1*Δ/Δ mutant followed by i.p. injection of 200 μg Gr-1 antibody, isotype control, or PBS alone on days 12, 14, and 16 postinoculation. Mice were sacrificed 24 h after the last treatment for collection of bone marrow, peritoneal lavage fluid (PLF), and spleens. Cells from each organ/site were incubated with fluorophore-conjugated anti-Gr-1 antibody and analyzed by flow cytometry to confirm depletion of Gr-1^+^ cells. Numbers adjacent to the gray areas (A) and represented in the graph (B) are the percentages of Gr-1^+^ cells in the bone marrow, PLF, and spleens of control mice (PBS alone) compared to those in mice treated with isotype control and anti-Gr-1 antibodies. Data were analyzed using Student’s *t* test. ** *P* < 0.01; * *P* < 0.05. Download FIG S2, TIF file, 6.5 MB. Copyright © 2021 Lilly et al. 2021 Lilly et al. https://creativecommons.org/licenses/by/4.0/ This content is distributed under the terms of the Creative Commons Attribution 4.0 International license.

### Relative role of leukocyte populations in mediating trained innate immune protection induced by i.v. vaccination followed by i.p. or i.v. lethal challenge.

**FIG 2:**
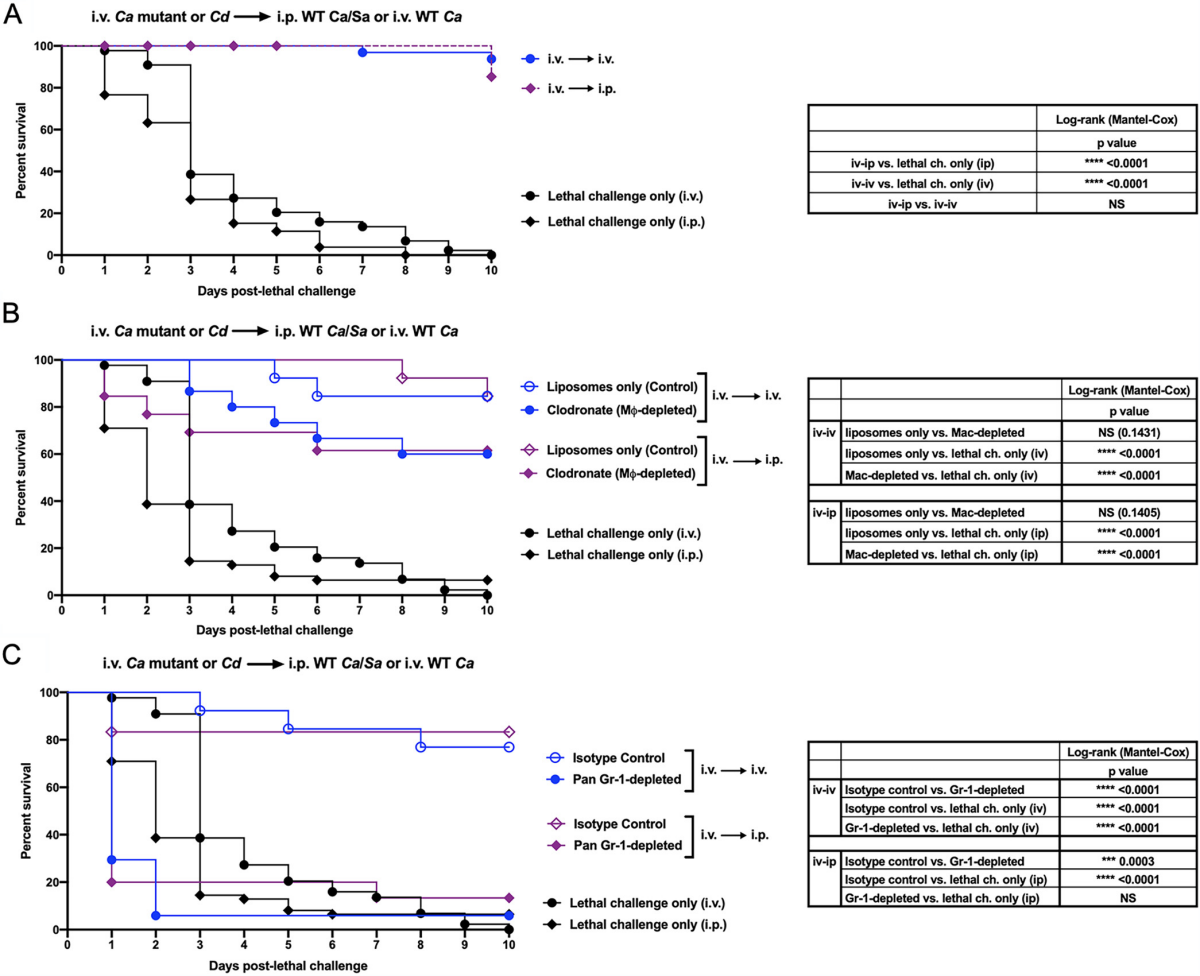
Relative role of leukocyte populations in mediating trained innate immune protection induced by intravenous vaccination followed by intraperitoneal or intravenous challenge. (A) Mice (*n* = 10/group) were given i.v. primary challenge (vaccination) of the C. albicans *efg1*Δ/Δ *cph1*Δ/Δ mutant or C. dubliniensis 14 days prior to i.p. (C. albicans/S. aureus) or i.v. (C. albicans) lethal challenge. (B) Role of macrophages. Mice (*n* = 10/group) given the primary challenge (i.v.) 14 days prior were injected i.p. with liposome-encapsulated clodronate (which results in ∼90% depletion of resident macrophages) or liposomes only 1 day prior to lethal challenge (i.p. or i.v.). (C) Role of Gr-1^+^ cells. Mice (*n* = 10/group) given the primary challenge (i.v.) 14 days prior were injected i.p. with 200 μg anti-Gr-1 (Ly6G/C) antibody to deplete Gr-1^+^ leukocytes or isotype control antibody 48 h prior to and 2 h after lethal challenge (i.p. or i.v.). Antibodies were given every 2 days to the remaining live animals for the duration of the study. For all studies, mice were monitored for 10 days post-lethal challenge. Animals receiving no primary challenge served as the positive (lethal) controls. Results shown are cumulative of 6 independent experiments. Data were analyzed using the log-rank (Mantel-Cox) test. Actual *P values* are listed in the tables. ****, *P* < 0.0001; ***, *P* < 0.001; **, *P* < 0.01; *, *P* < 0.05.

We next sought to determine if an i.v. route of vaccination could protect against an i.p. or i.v. lethal challenge. For these studies, mice were vaccinated i.v. with a low-virulence *Candida* strain (*Cd* or C. albicans *efg1*Δ/Δ *cph1*Δ/Δ double-null mutant) followed 14 days later by lethal challenge i.p. (*Ca*/*Sa*) or i.v. (*Ca* alone). Primary i.v. vaccination resulted in significant survival (80 to 90%) against both i.p. and i.v. lethal challenge (Fig. 2A) (*P* < 0.0001).

Similar cellular depletion experiments were subsequently conducted following the i.v. vaccination. Results show no significant differences in survival between animals treated with liposomal clodronate and liposome vehicle alone prior to i.p. (*Ca*/*Sa*) or i.v. (*Ca*) lethal challenge (Fig. 2B). In contrast, vaccinated mice administered anti-Gr-1 antibodies, but not isotype control antibodies, and challenged i.p. or i.v. showed significantly reduced survival (Fig. 2C).

**FIG 3:**
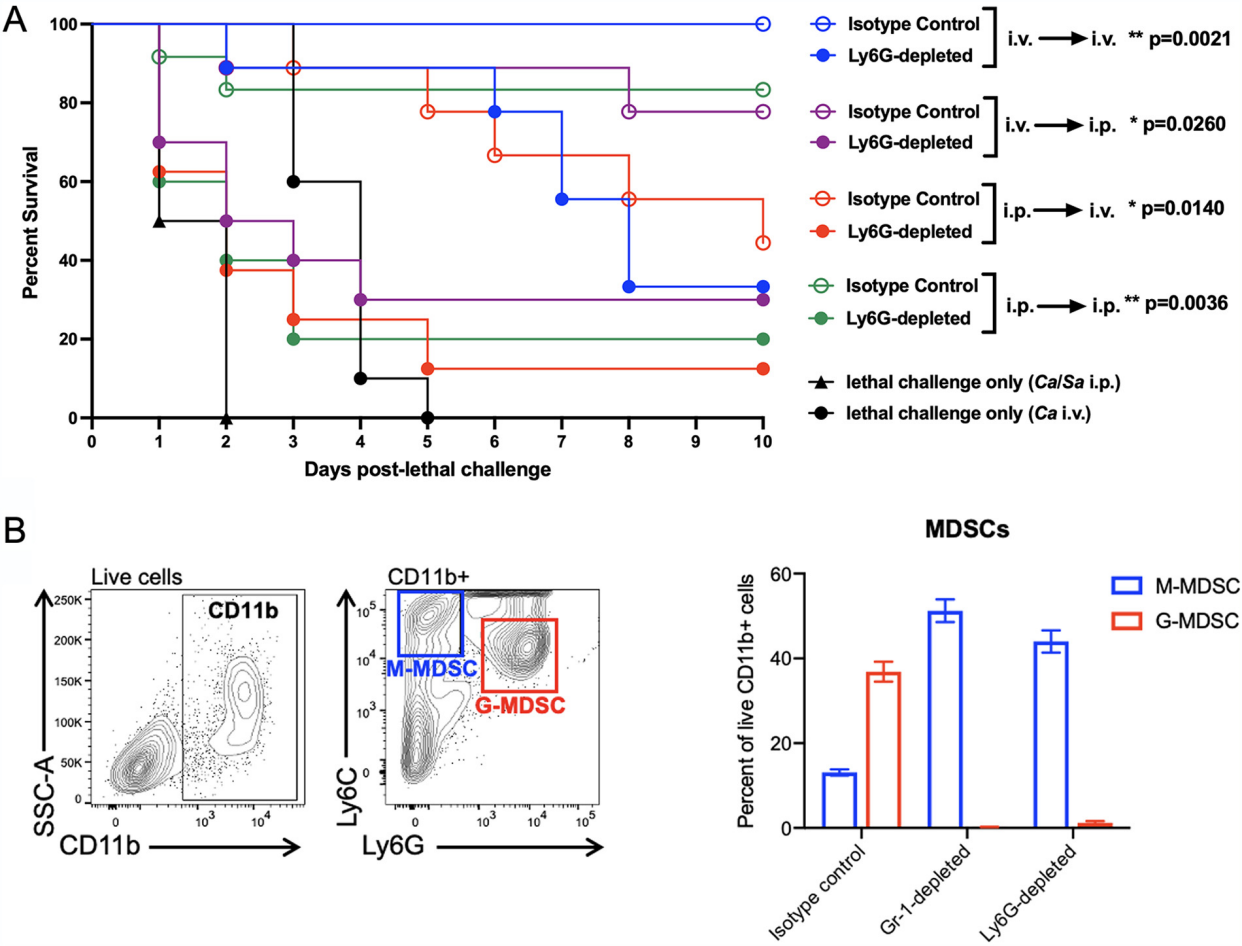
Confirmation of the role of the Ly6G^+^ subset of Gr-1^+^ leukocytes in mediating trained innate immune protection in all models. (A) Mice (*n* = 10/group) given the primary challenge (i.v. or i.p.) of the C. albicans *efg1*Δ/Δ *cph1*Δ/Δ mutant 14 days prior were injected i.p. with 200 μg anti-Ly6G antibody to deplete PMNs/G-MDSCs or isotype control antibody 48 h prior to and 2 h after i.p. (C. albicans/S. aureus) or i.v. (C. albicans) lethal challenge. Antibodies were given every 2 days to the remaining live animals for the duration of the study. For all studies, mice were monitored for 10 days post-lethal challenge. Animals receiving no primary challenge served as the positive (lethal) controls. Results shown are cumulative of 2 independent experiments. Isotype control and Ly6G-depleted groups were analyzed using the log-rank (Mantel-Cox) test. Actual *P* values are shown in the graph. **, *P* < 0.01; *, *P* < 0.05. (B) Mice (*n* = 3/group) were inoculated i.v. with the C. albicans *efg1*Δ/Δ *cph1*Δ/Δ mutant followed by i.p. injection of 200 μg anti-Gr-1, anti-Ly6G, or isotype control antibody on days 12, 14, and 16 postinoculation. Mice were sacrificed 24 h after the last treatment for collection of spleens and depletion of M-MDSCs (CD11b^+^ Ly6C^hi^ Ly6G^−^) and G-MDSCs (CD11b^+^ Ly6C^lo^ Ly6G^+^) in each group were assessed by flow cytometry.

Gr-1 is a surface receptor and consists of Ly-6G and Ly-6C antigens. Therefore, Gr-1 antibody depletion will affect both Ly6G^+^ granulocytic cells (neutrophils and G-MDSCs) and Ly6C^+^ monocytic cells (monocytes and M-MDSCs). Therefore, according to our hypothesis, it was important to confirm that the protection in each model was being mediated specifically/predominantly by granulocytic Gr-1^+^ cells as opposed to monocytes. For this, vaccinated animals were treated with anti-Ly6G antibodies similar to the Gr-1^+^ cellular depletion for each model just prior to and during the lethal challenge period. As anticipated, in each model, depletion of Ly6G^+^ cells abrogated the vaccination-mediated protection as evidenced by significantly enhanced mortality compared to that of the animals administered the isotype control antibody (Fig. 3A). Flow cytometry analysis of MDSC populations in the blood and spleens of vaccinated mice confirmed that both Ly6G and Gr-1 antibody treatment specifically depleted G-MDSCs (Fig. 3B and data not shown).

### Evidence that protection against i.v. lethal challenge induced by i.p. or i.v. vaccination is associated with reduced sepsis.

### (i) Hypothermia and sepsis scores.

**FIG 4:**
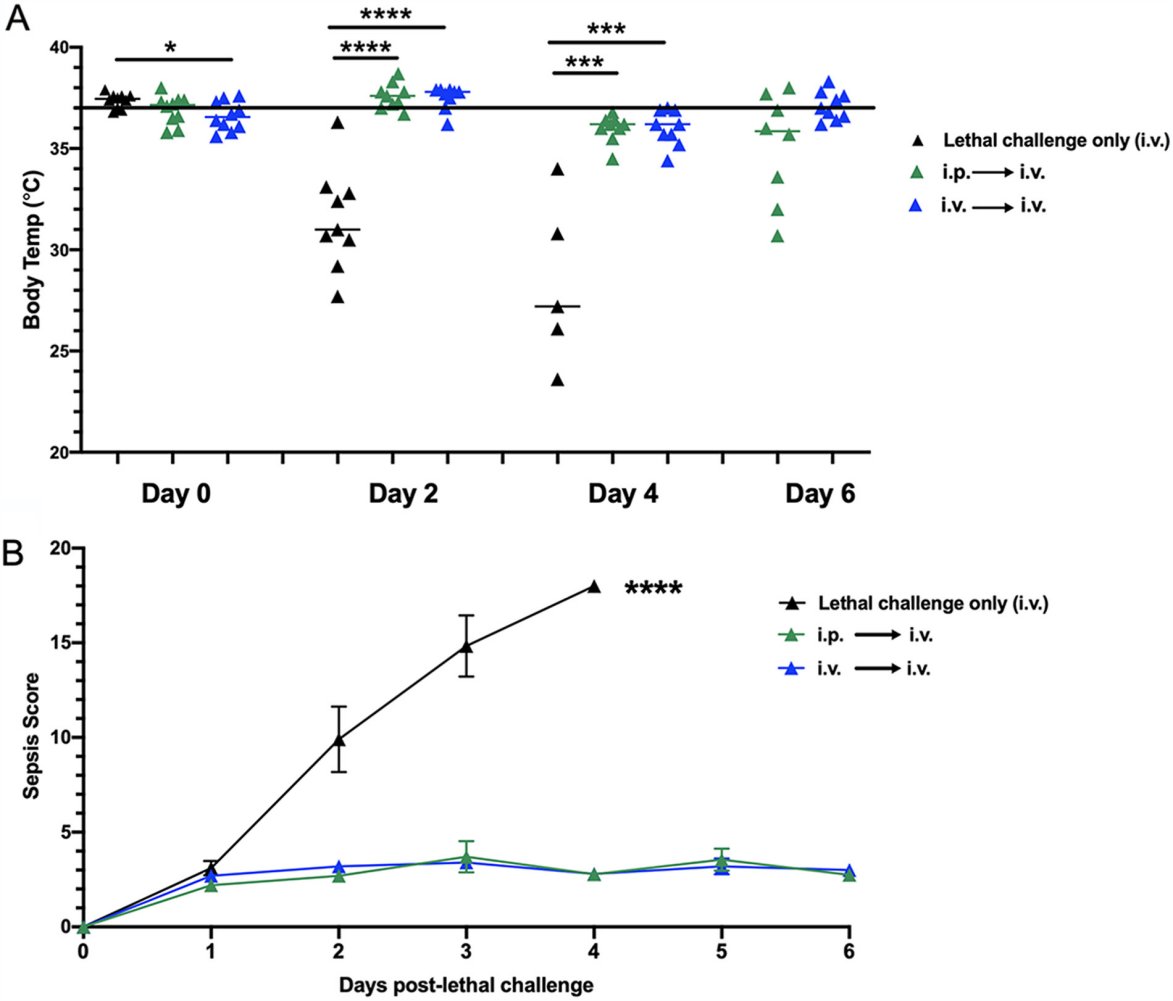
Effect of intraperitoneal or intravenous vaccination with avirulent *Candida* strains on body temperature and sepsis scores following intravenous C. albicans infection. (A) Hypothermia. Mice (*n* = 5/group) were given primary challenge (i.p. or i.v. vaccination) of the C. albicans *efg1*Δ/Δ *cph1*Δ/Δ mutant 14 days prior to i.v. (C. albicans) lethal challenge. Unvaccinated mice (lethal challenge only) were included as controls. Body temperatures were recorded on day 0 just prior to lethal challenge and 2, 4, and 6 days post-lethal challenge. (B) Sepsis scoring. Mice (*n* = 10/group) were observed and scored daily for morbidity (hunched posture, inactivity, and ruffled fur) using the Mouse Clinical Assessment Score for Sepsis (M-CASS) (13) starting at day 0 and continuing for 6 days after lethal intravenous challenge. Results shown are cumulative of 3 independent experiments. Data were analyzed using the Mann-Whitney U test (body temperatures) or Student’s *t* test (sepsis scoring). ****, *P* < 0.0001; ***, *P* < 0.001; *, *P* < 0.05.

Previous work in our laboratory demonstrated that during polymicrobial intra-abdominal infections (IAI) with *Ca* and *Sa*, heightened inflammation and hypothermia, but not microbial burden, were associated with mortality (3, 4). To investigate this in the lethal i.v. challenge model, core body temperatures of avirulent *Candida* strain-vaccinated (i.p. or i.v.) mice were compared to those in control lethal challenge mice at day 0 just prior to i.v. lethal challenge and days 2, 4, and 6 post-lethal challenge. In lethal challenge control animals, hypothermia was observed as early as day 2 (*P* < 0.0001) and continued at day 4 (*P* < 0.001) before they all succumbed (Fig. 4). In vaccinated (i.v. or i.p.) mice just prior to lethal challenge, body temperatures were slightly below those of unvaccinated mice (36.6°C versus 37.4°C, respectively) (*P* = 0.012). Through the observation period temperatures in vaccinated mice varied slightly up and then down by day 4. By day 6, temperatures in the animals given primary i.v. vaccination appeared to return to normal, while temperatures in i.p.-vaccinated mice had a wider range (Fig. 4A). Repeated-measures analysis supported the temperature continuum (vaccinated i.p./i.v., *P* = 0.03; vaccinated i.v./i.v., *P* = 0.0012; unvaccinated, *P* = 0.0002). A portion of the i.p.-vaccinated animals (30 to 40%) were humanely sacrificed before the day 10 endpoint due to behaviors indicative of a neurological condition described previously (9).

As another measure of sepsis, vaccinated (i.p. or i.v.) mice and unvaccinated control mice receiving lethal i.v. challenge only were monitored daily for morbidity (hunched posture, inactivity, and ruffled fur) and scored using the Mouse Clinical Assessment Score for Sepsis (M-CASS) system (13). Between days 2 and 4 post-lethal challenge, vaccinated animals had significantly lower M-CASS scores than did lethal control mice (*P* < 0.0001) that all succumbed by day 4. Thereafter, the M-CASS scores in vaccinated mice continued to be low through the 6-day observation period (Fig. 4B). No differences were observed in sepsis scores between i.p.- and i.v.-vaccinated mice throughout despite the ethical sacrifice of the majority of i.p.-vaccinated mice.

### (ii) Systemic cytokine production.

**FIG 5:**
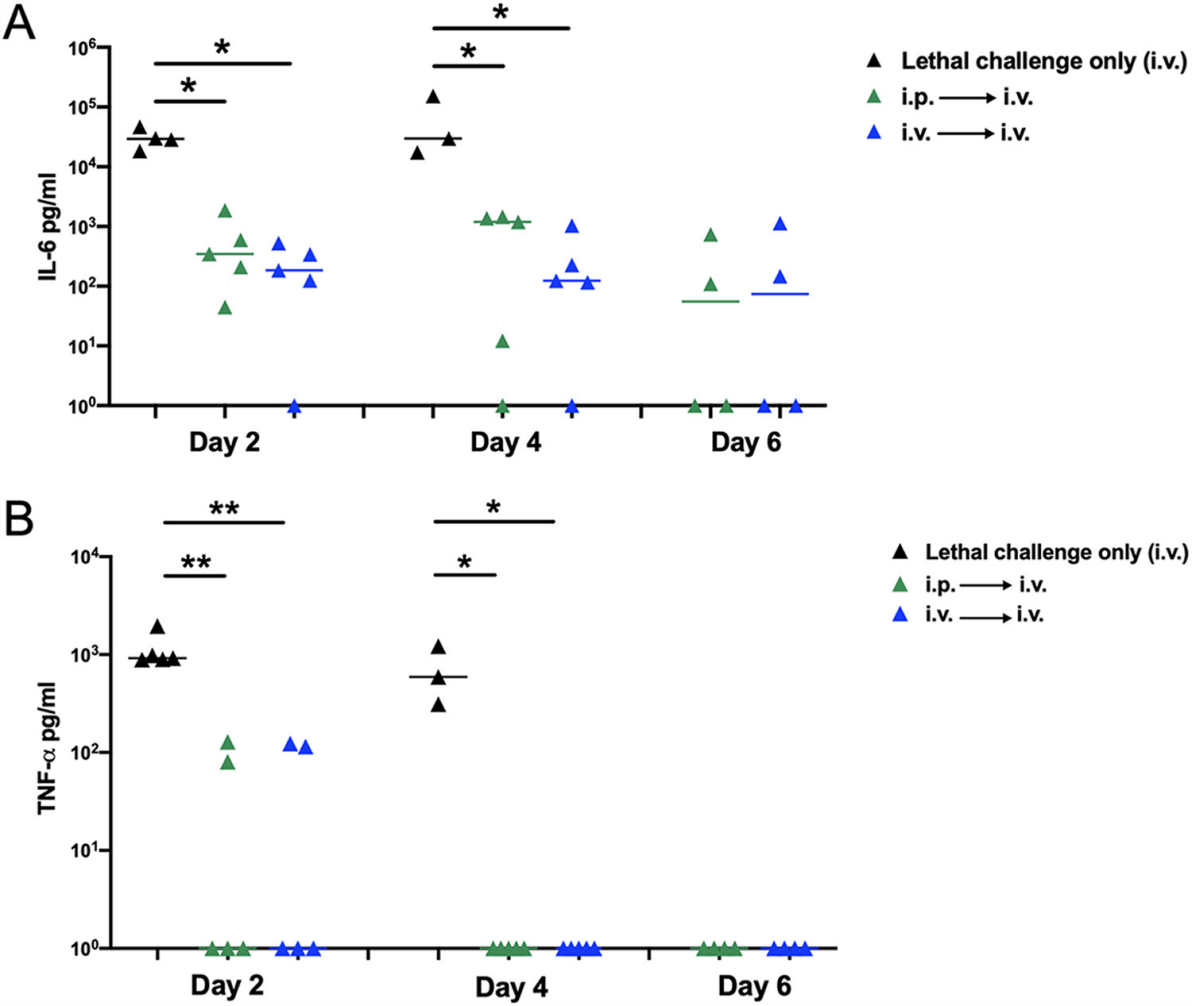
Effect of intraperitoneal or intravenous vaccination with avirulent *Candida* strains on proinflammatory cytokine production following intravenous C. albicans infection. Mice (*n* = 5/group) were given primary challenge (i.p. or i.v. vaccination) of the C. albicans *efg1*Δ/Δ *cph1*Δ/Δ mutant 14 days prior to i.v. (C. albicans) lethal challenge. Unvaccinated mice (lethal challenge only) were included as controls. Animals were sacrificed for serum collection on days 2, 4, and 6 post-lethal challenge. Serum was analyzed for IL-6 (A) and TNF-α (B) by ELISA. Data are cumulative of 2 independent experiments and were analyzed using the Mann-Whitney U test. **, *P* < 0.01; *, *P* < 0.05.

We previously demonstrated that during polymicrobial intra-abdominal infections (IAI) with *Ca* and *Sa*, heightened proinflammatory cytokine levels (IL-6, IL-1β, and TNF-α) were closely associated with mortality (4, 5). A similar hyperinflammatory response is also well documented for *Candida* bloodstream infections leading to sepsis (14, 15). To determine the effects of i.p. or i.v. vaccination on systemic proinflammatory cytokines, we examined serum IL-6 and TNF-α at days 2, 4, and 6 post-lethal challenge in vaccinated (i.p. or i.v.) and lethal control animals. Both IL-6 (Fig. 5A) and TNF-α (Fig. 5B) levels were significantly reduced in i.p.- and i.v.-vaccinated animals compared to those in lethal control animals at days 2 and 4. Levels of both cytokines continued to be reduced at day 6 in vaccinated mice, when lethal control mice had expired. No differences in cytokine levels were observed between i.p.- and i.v.-vaccinated mice, again despite the forced delayed mortality in i.p.-vaccinated mice.

### (iii) Organ fungal burden.

**FIG 6:**
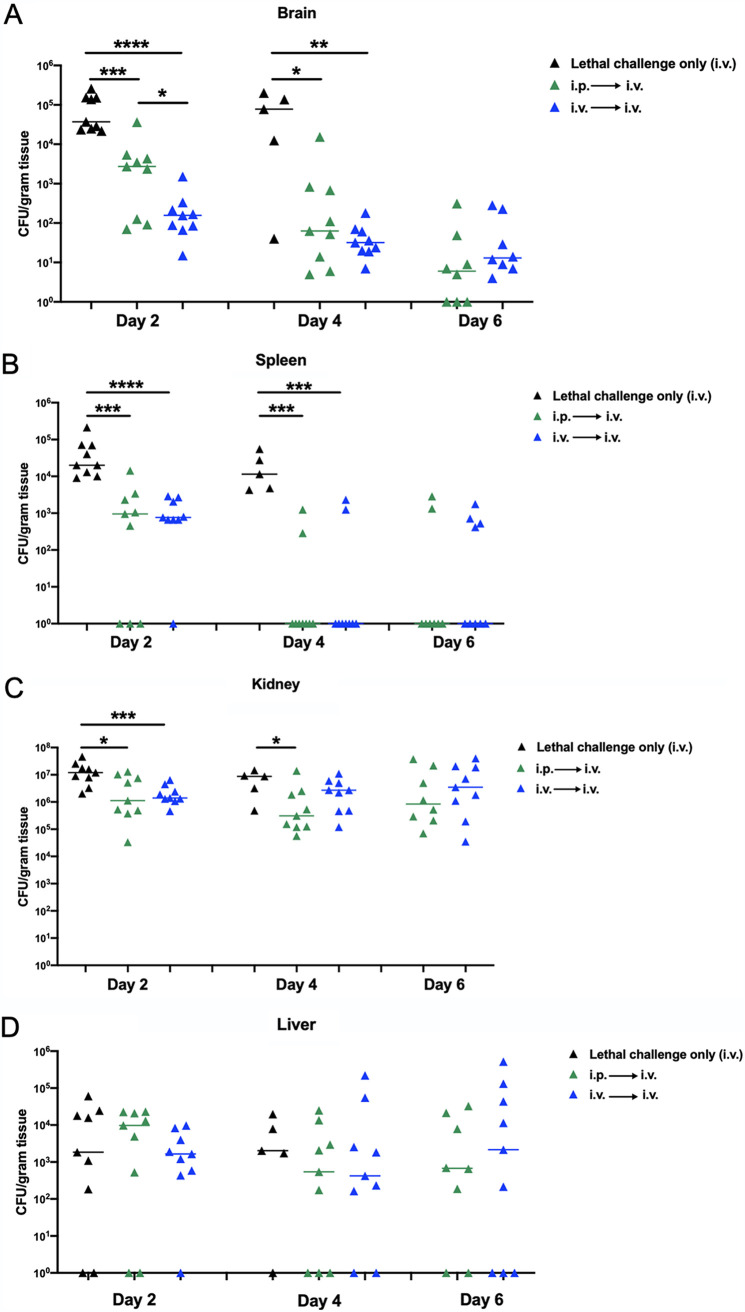
Effect of intraperitoneal or intravenous vaccination with avirulent *Candida* strains on target organ fungal burden following intravenous C. albicans infection. Mice (*n* = 5/group) were given primary challenge (i.p. or i.v. vaccination) of the C. albicans *efg1*Δ/Δ *cph1*Δ/Δ mutant 14 days prior to i.v. (C. albicans) lethal challenge. Unvaccinated mice (lethal challenge only) were included as controls. Animals were sacrificed on days 2, 4, and 6 post-lethal challenge and assessed for brain (A), spleen (B), kidney (C), and liver (D) fungal burdens. Data are cumulative of 2 independent experiments and were analyzed using the Mann-Whitney U test. ****, *P* < 0.0001; ***, *P* < 0.001; **, *P* < 0.01; *, *P* < 0.05.

Organ fungal burden (brain, spleen, kidney, and liver) was determined at days 2, 4, and 6 post-lethal challenge. Results in Fig. 6 show that at days 2 and 4 post-i.v. lethal challenge, significant reductions in CFU were observed in i.p.- and i.v.-vaccinated animals compared to lethal controls in all organs with the exception of the liver. Overall, reductions of CFU in vaccinated mice were greatest in the brain, followed by the spleen and kidney. Notably, the CFU in the kidney were 2 logs higher than in the brain or spleen. After day 4, only vaccinated mice were monitored, as unvaccinated mice had all succumbed. Organ fungal burdens were similar for i.v.- and i.p.-vaccinated mice throughout the 6 days, with one exception: brain CFU were significantly reduced at day 2 in i.v.-vaccinated animals compared to those in i.p.-vaccinated animals (*P* = 0.04) (Fig. 6A), which were ultimately humanely sacrificed thereafter.

## DISCUSSION

We previously reported that primary i.p. challenge or vaccination of mice with low-virulence/attenuated *Candida* species (i.e., C. dubliniensis, C. auris, C. glabrata, and C. albicans *efg1*Δ/Δ *cph1*Δ/Δ) protects against i.p. or i.v. lethal fungal challenge (sepsis), with trained innate protection against lethal *Ca/Sa* IAI (i.p.) mediated by long-lived Gr-1^+^ leukocytes (6, 9). These findings prompted us to investigate trained innate protection in several permutations of the models using different routes of vaccination and lethal challenge, the role of sepsis in each, and identification of the cells involved in protection.

Highly significant in the present study is that i.p. or i.v. vaccination with low-virulence *Candida* species protects against i.p. or i.v. lethal challenge. Mortality in both *Ca*/*Sa* IAI (i.p.) and *Ca* BSI (i.v.) is attributable to sepsis as evidenced by hypothermia observed in these animals as well as significantly high M-CASS scores and increased systemic proinflammatory cytokine production (IL-6 and TNF-α) compared to those in vaccinated mice before they succumb. In all cases, Gr-1^+^ leukocytes, and specifically/predominantly Ly6G^+^ leukocytes, are responsible for trained innate protection against lethal sepsis. Considering the short life span (24 h) of polymorphonuclear neutrophils (PMNs), these results continue to support the contention that these long-lived Gr-1^+^ cells are Ly6G^+^ G-MDSCs, with similar abrogation of protection observed with Gr-1 or Ly6G antibody-mediated cellular depletion. While this still needs to be confirmed mechanistically, MDSCs are known to be induced in the bone marrow, and we previously reported on the ability of various *Candida* species to infiltrate bone marrow upon vaccination, with the level of infiltration correlating with protection (9). We also confirmed that both vaccine strains (C. dubliniensis and C. albicans *efg1*Δ/Δ *cph1*Δ/Δ) access the bone marrow following either i.p. or i.v. inoculation as early as 24 h but are cleared by day 14, the exception being low-level residual CFU in a small percentage of mice following i.v. inoculation. Therefore, there is a possibility that a sustained presence of avirulent fungal burden in the bone marrow of these mice results in “priming” (continued activation state) as opposed to “training” (cells return to unactivated baseline state) (16). However, the consistency in protection observed in each group argues against this playing a major role. If priming were responsible for protection, animals that cleared the vaccine strain from the bone marrow (80% of mice) would not be protected following lethal challenge. Instead, we observed ∼90% protection following i.v. vaccination against either i.p. or i.v. challenge, strongly supporting the contention that innate training is responsible. Other evidence supporting training of Ly6G^+^ MDSCs (as opposed to continued activation of inflammatory Ly6G^+^ PMNs) is the reduction in proinflammatory cytokines in all mice concomitant with protection irrespective of route of vaccination.

Consistent with our previous findings, monocytes/macrophages (trained or otherwise) played no role, as demonstrated by the lack of abrogation of protection following liposomal clodronate treatment. A slight reduction in survival was observed in the i.v. vaccination followed by i.p. lethal challenge, but this was not statistically significant compared to results for control mice receiving liposomes only. Nevertheless, we cannot exclude some role for monocytes/macrophages in the i.v. vaccination/i.p. lethal challenge model. In addition, our laboratory previously confirmed no role for T or B cells in the i.p. vaccination/i.p. lethal challenge *Ca*/*Sa* IAI model via the use of RAG1^−/−^ mice (6). Results showing the complete abrogation of protection following both Gr-1^+^ and Ly6G^+^ cell depletions (which also resulted in the depletion of putative G-MDSCs [Fig. 3B]) in all the other model permutations (i.p. vaccination/i.v. lethal challenge, i.v. vaccination/i.p. lethal challenge, and i.v. vaccination/i.v. lethal challenge) continue to argue against any role for T cells, B cells, macrophages, or monocytes. Hence, our results showing a strong role for trained Ly6G^+^ Gr-1^+^ granulocytes in protection continue to build on this novel form of trained innate immunity that inhibits sepsis as the primary protective mechanism. We have termed this novel TII “trained tolerogenic immunity” (17) as opposed to conventional TII, which involves trained monocytes/macrophages with enhanced immune responsiveness (18, 19). It will be interesting to determine the timing for induction of this trained tolerogenic immunity in the bone marrow postvaccination. Preliminary evidence from our laboratory suggests that it may be 7 days or less.

These unique and novel findings appear to conflict with a seminal TII study by Quintin and colleagues documenting C. albicans functional reprogramming of monocytes *in vivo* following infection priming (20). These studies employed a similar model of i.v. sublethal primary challenge/i.v. lethal challenge using C. albicans and similarly showed the lack of a role for T and B cells in RAG1^−/−^ mice. To investigate a role for monocytes/macrophages, the authors demonstrated that protection against reinfection was abrogated in CCR2^−/−^ mice, which are defective in monocyte recruitment/trafficking. For our studies, using a more direct approach of clodronate treatment, which depletes most tissue macrophage populations as well as circulating Ly6C^+^ monocytes (21), failed to abrogate protection, whereas depletion of Ly6G^+^ cells significantly abrogated protection. The discrepancy in the findings of these two studies may be partially explained by the less explored role of CCR2 in controlling Gr-1^+^ neutrophil migration during sepsis or models of acute inflammation (22, 23). Therefore, CCR2 deficiency could potentially also impact Gr-1^+^ neutrophil and/or MDSC recruitment considering i.v. C. albicans infection results in sepsis. Hence, it is quite possible that the results of both our study and the study by Quintin and colleagues were reflective of protection mediated by trained Gr-1^+^ MDSC-mediated inhibition of sepsis rather than trained monocyte/macrophage antifungal activity.

The trained innate antisepsis immunity we describe in all the model permutations of lethal sepsis offers the potential for a novel form of vaccination (immune-preventive) against candidemia via induction of Ly6G^+^ Gr-1^+^ G-MDSCs. In each of the models (i.p. and i.v. vaccination followed by i.p. or i.v. lethal challenge), significant reductions in hypothermia, sepsis scoring, and serum cytokines correlated with enhanced survival. We also observed significantly reduced fungal burden in target organs, indicating improved antifungal responses that could directly involve MDSCs. Previous studies have demonstrated that Gr-1^+^ MDSCs have antifungal activity *in vitro* (8). We hypothesize that the vaccination-induced TII protects against sepsis, thereby allowing time for the innate/adaptive immune response to ultimately eliminate the pathogen.

An interesting observation in the i.p. vaccination route against i.v. lethal challenge is the delayed mortality compared to almost 100% survival in i.v. vaccination against i.v. lethal challenge. In these cases, ethical sacrifice of mice was conducted before the study endpoint due to a neurological condition with behaviors associated with encephalitis (chronic circling). Evidence suggests that sepsis did not prompt this ethical sacrifice, since only slight hypothermia was observed, with low sepsis scores recorded until ethical sacrifice. Instead, the neurological condition appears related to brain fungal burden, which was increased 2 days post-i.v. lethal challenge, followed by considerable variability at 4 days compared to i.v.-vaccinated mice similarly given the i.v. lethal challenge. Notwithstanding of the neurological condition, the animals appeared healthy. This relationship was not shown for fungal burden in the other organs (spleen, kidney, and liver), as organ CFU were similar between the i.p. vaccination/i.v. lethal challenge and i.v. vaccination/i.v. lethal challenge models. Of note as well, mice depleted of Gr-1^+^ cells succumbed quickly to sepsis, never reaching the ethical sacrifice point. This supports the hypothesis that sepsis was not a factor in the neurological condition and that those mice had indeed been protected against the lethal sepsis.

In terms of fungal burden, kidney and liver burdens were maintained at high levels in both protected and nonprotected control animals. This suggests that kidney and liver burdens are not related to sepsis and do not need to be controlled/reduced for survival. The same is probably true for the spleen and brain despite the reduced CFU correlation with protection. The reduced spleen burden is consistent with the spleen’s role in filtering antigens from the blood and the systemic nature of the lethal infection. There are many reports of high kidney burden in i.v. challenge candidemia models (24–26), although this usually reflects simply a parameter of infection. Our study places into question what role the kidney burden has in mortality versus survival in these challenge models. It is interesting to consider that since Gr-1^+^ cells are responsible for protection against i.p. or i.v. lethal challenge, the higher brain and spleen fungal burdens in lethal challenge control mice than in vaccinated mice suggest some level of antifungal activity by Gr-1^+^ PMNs in the brains and spleens of mice protected against sepsis.

It is also interesting to consider whether i.p. or i.v. vaccination by low-virulence/attenuated *Candida* species can protect against sepsis produced by other pathogens/insults. We predict based on the innate training that protection would be observed against several initiators of sepsis. The reverse should be considered as well: can live attenuated childhood vaccines protect against *Ca* or *Ca*/*Sa* sepsis via induction of MDSCs? Current studies in our laboratory are focusing on whether the live attenuated MMR vaccine can induce MDSCs that would protect against i.p. or i.v. lethal challenge with *Candida*. This general concept is also being investigated with the live attenuated bacillus Calmette-Guérin (BCG) vaccine, which also infiltrates bone marrow and is known to induce trained innate macrophages (27). In these cases, BCG is being evaluated for enhanced nonspecific immunity against unrelated infections.

These concepts are also now being investigated in COVID-19 research. Clinical trials are ongoing using BCG and measles-mumps-rubella (MMR) vaccines to mitigate sequelae of COVID-19. For the BCG vaccine the concept is to induce TII to enhance immunity against severe acute respiratory syndrome coronavirus 2 (SARS-CoV-2). For the MMR vaccine the concept and major endpoint of the clinical trials are to induce trained MDSCs to mitigate COVID-associated sepsis or sepsis by other insults. There are considerable anecdotal and circumstantial clinical data that support a role for the MMR vaccine for both cross-reactivity of mumps, measles, and/or rubella antibodies against SARS-CoV-2 infection and mitigation of severe symptoms (i.e., sepsis) associated with COVID-19 (28, 29). Hence, live attenuated vaccines or naturally attenuated microbial species may serve as a source for trained innate immunity that may be beneficial as nonspecific immune preventives for infectious disease via inhibition of sepsis or enhanced responsiveness.

## MATERIALS AND METHODS

### Mice.

Female Swiss Webster mice, 5 to 7 weeks of age, were purchased from Charles River Laboratories. Animals were housed and handled according to institutionally recommended guidelines. All experiments involving animals were approved by the Tulane University School of Medicine Institutional Animal Care and Use Committee.

### Strains and growth conditions.

C. albicans strain DAY185, a prototrophic derivative of SC5314, was a gift from Aaron Mitchell (Carnegie Mellon University, Pittsburgh, PA). The C. albicans *efg1*Δ/Δ *cph1*Δ/Δ mutant strain (parental strain: SC5314-CAI4) was kindly provided by Glen Palmer (University of Tennessee Health Sciences Center, Memphis, TN). The C. dubliniensis wild-type strain (Wü284) was kindly provided by Gary Moran (Trinity College, Dublin, Ireland). Frozen stocks were maintained at −80°C and streaked onto yeast peptone dextrose (YPD) agar prior to use. A single colony was transferred to 10 ml YPD broth and shaken at 30°C for 12 to 18 h. The methicillin-resistant *Sa* strain NRS383 used in i.p. lethal challenge experiments was obtained from the Network on Antimicrobial Resistance in Staphylococcus aureus (NARSA) data bank. Frozen stocks were maintained at −80°C and streaked onto Trypticase soy agar (TSA) prior to use. A single colony was transferred to 10 ml Trypticase soy broth (TSB) and shaken at 37°C overnight. On the following day, the overnight culture was diluted 1:100 in fresh TSB and shaken at 37°C for 3 h until the culture reached the log phase of growth. Prior to inoculation, organisms were washed 3 times by centrifugation in sterile phosphate-buffered saline (PBS; pH 7.4), counted on a hemocytometer, and diluted in sterile PBS to prepare standardized inocula.

### Vaccination.

Groups (*n* =10) of 6-week-old outbred Swiss Webster mice were injected with avirulent *Candida* strains (C. dubliniensis or C. albicans *efg1*Δ/Δ *cph1*Δ/Δ mutant strain) intraperitoneally (1.75 × 10^7^/mouse) in a volume of 200 μl or intravenously (1 × 10^5^/mouse) in a volume of 100 μl via tail vein injection 14 days prior to lethal challenge. We monitored clearance of vaccine strains from the bone marrow (site of initiation of innate training) following vaccination, with the majority of mice showing undetectable burden by day 14 following i.p. inoculation and only low-level burden in a small percentage of mice following i.v. inoculation (Fig. S3).

10.1128/mBio.02548-21.3 FIG S3 Bone marrow CFU following intraperitoneal or intravenous vaccination with C. dubliniensis or the C. albicans *efg1*Δ/Δ *cph1*Δ/Δ double-null mutant. Mice (*n *=* *10/group) were sacrificed on days 7 and 14 following challenge with C. albicans *efg1*Δ/Δ *cph1*Δ/Δ (i.v. or i.p.) or C. dubliniensis (i.p.), and femoral bone marrow was isolated and assessed for fungal burden. Results are expressed as CFU per milliliter bone marrow cell suspension. Data are cumulative of 2 separate experiments. Download FIG S3, TIF file, 0.8 MB. Copyright © 2021 Lilly et al. 2021 Lilly et al. https://creativecommons.org/licenses/by/4.0/ This content is distributed under the terms of the Creative Commons Attribution 4.0 International license.

### Murine model of fungal-bacterial intra-abdominal infection.

Mice were injected i.p. with a lethal challenge of *Ca* (1.75 × 10^7^/mouse) and *Sa* (8 × 10^7^/mouse) in a volume of 200 μl and observed for morbidity (hunched posture, inactivity, and ruffled fur) and mortality up to 10 days after rechallenge.

### Murine model of bloodstream infection.

Mice were given a lethal challenge (i.v.) of *Ca* DAY185 (1 × 10^5^/mouse) via tail vein injection (100 μl) 14 days after vaccination (i.p. or i.v.). Mice were observed and scored daily for morbidity (hunched posture, inactivity, and ruffled fur) and mortality using the Mouse Clinical Assessment Score for Sepsis (M-CASS) (13) for 10 days after lethal i.v. challenge. Control mice received the lethal i.v. challenge only. In some studies, groups of 5 mice were sacrificed on days 2, 4, and 6 post-lethal challenge to assess organ fungal burden and serum cytokine levels. Rectal temperature was measured using a three-quarter-inch rectal probe (ThermoWorks, American Fork, UT) at day 0 and just prior to sacrifice. For this, mice were anesthetized with 3% isoflurane followed by insertion of the probe into the rectum.

### Organ fungal burden.

The brain, spleen, liver, and kidneys were removed from each mouse immediately following sacrifice. Each organ was weighed and homogenized in 1 ml PBS using the PRO 200 tissue homogenizer (PRO Scientific, Oxford, CT). For bone marrow isolation, femurs were isolated from each mouse and each bone was flushed with 5 to 10 ml cold PBS using a 27_1/2_-gauge needle. Red blood cells (RBCs) were lysed in 1× RBC lysis buffer (Thermo Fisher), and cells were resuspended in 1 ml sterile PBS. Fungal burden in brain, spleen, liver, and kidneys was enumerated by serial dilution plating of homogenates onto YPD agar containing 20 μg/ml nafcillin and 2 μg/ml vancomycin using the drop plate method (30). Plates were incubated overnight at 37°C. All CFU counts were expressed as the number of CFU per gram tissue. Bone marrow isolated cells were plated neat. Plates were incubated overnight at 37°C. CFU counts were expressed as the number of CFU per gram tissue or CFU per milliliter bone marrow isolated cells.

### Serum cytokine analysis.

Following sacrifice, whole blood was collected by cardiac puncture and allowed to sit overnight at 4°C. Serum was separated by centrifugation at 10,000 × *g* for 2 min, aliquoted, and stored at −80°C until analysis. Concentrations of interleukin-6 (IL-6) and tumor necrosis factor alpha (TNF-α) were determined by single-plex enzyme-linked immunosorbent assays (ELISAs; BioLegend, San Diego, CA).

### F4/80^+^ macrophage depletion.

Liposome-encapsulated clodronate and liposome vehicle (1 mg/mouse; Encapsula NanoSciences, Brentwood, TN) were injected i.p. in 200 μl 1 day prior to lethal challenge of animals with *Ca*/*Sa* (i.p.) *or Ca* alone (i.v.) as previously described (6). Depletion was confirmed by flow cytometry (Fig. S1).

### Gr-1^+^ Ly6G^+^ leukocyte depletion.

Mice were injected i.p. with either 200 μg rat anti-mouse Gr-1 (Ly6G/Ly6C), Ly6G, or rat IgG2A isotype control antibodies (Bio-X-Cell, Lebanon, NH) in 200 μl sterile PBS to systemically deplete PMNs/MDSCs 48 h prior to and 2 h after lethal challenge with *Ca*/*Sa* (i.p.) or *Ca* alone (i.v.). Injections were given every 2 days for the duration of the study. Depletion was confirmed by flow cytometry (Fig. S2).

### Flow cytometry.

To confirm depletion of various cell types, cells were collected from peritoneal lavage fluid, spleen, and/or bone marrow 24 h after the last liposomal clodronate or antibody (anti-Gr-1 or -Ly6G) treatment. Following red blood cell lysis, single cell suspensions were incubated with Fc block (BD Biosciences; catalog number 553142) for 10 min on ice and then a combination of the following fluorophore-conjugated antibodies for 30 min on ice in the dark: anti-Gr-1 (Ly6G/C; BD; catalog number 553128), Ly6G (BD; catalog number 560601), Ly6C (BD; catalog number 560592), CD11b (BD; catalog number 557672, and F4/80 (macrophages; eBioscience; catalog number 17-4801-80). All antibodies were diluted 1:100 in fluorescence-activated cell sorting (FACS) buffer (PBS with 2% fetal bovine serum and 5 mM EDTA). Fixable viability dye eFluor 506 (eBioscience; catalog number 65-0866-14; 1:100) was included to label dead cells. Following staining, cells were washed twice and fixed with 4% paraformaldehyde for 15 min on ice. Following fixation, cells were washed twice, resuspended in FACS buffer, and stored at 4°C in the dark until analysis. Unstained cells and UltraComp eBeads compensation beads (Invitrogen, Carlsbad, CA) stained with individual fluorophores were used to calculate compensation. Fluorescence-minus-one (FMO) controls were included as gating controls. Cells were collected on a BD LSRFortessa flow cytometer (BD Biosciences, San Jose, CA) and the data were analyzed using FlowJo software (FlowJo, LLC, Ashland, OR).

### Statistics.

Survival curves were compared using the log-rank (Mantel-Cox) test. Organ fungal burden, body temperatures, and serum cytokine levels were analyzed using the Mann-Whitney U test. M-CASS sepsis scores were analyzed by Student’s *t* test. For cellular depletion studies, percentages of positive cells in each group were compared using Student’s *t* test. Significant differences were defined at a *P *value of *<*0.05. All statistical analyses were performed using Prism software (GraphPad, San Diego, CA).
