# Supplementary figures and images for: Trained Innate Immunity Induced by Vaccination with Low-Virulence Candida Species Mediates Protection against Several Forms of Fungal Sepsis via Ly6G+ Gr-1+ Leukocytes
Source: mBio. 2021 Oct 19;12(5):e02548-21. doi: 10.1128/mBio.02548-21 (PMC8524338; doi:10.1128/mBio.02548-21)

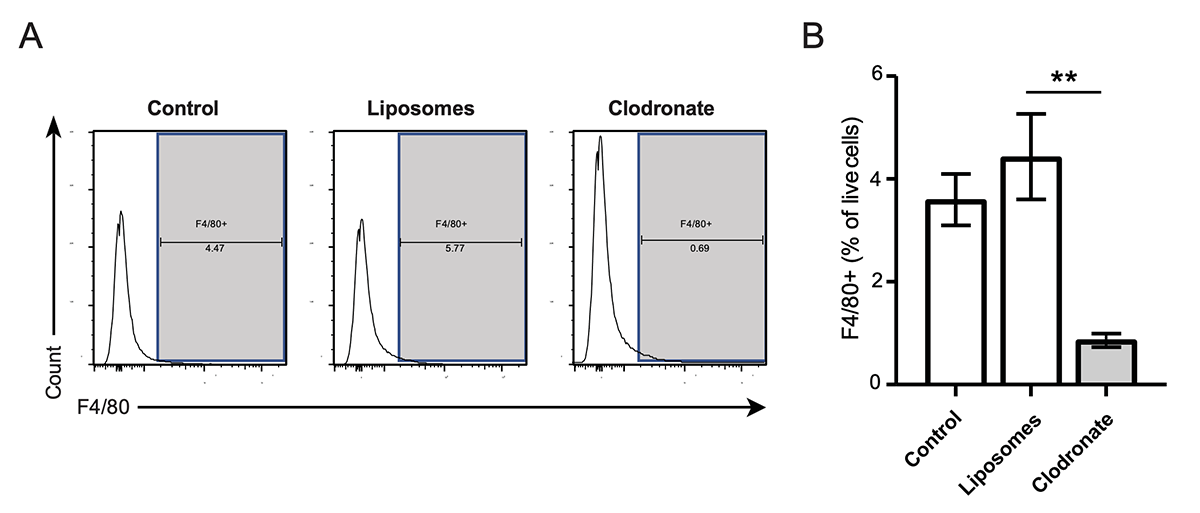

Supplement: FIG S1 [file mbio.02548-21-sf001.tif]

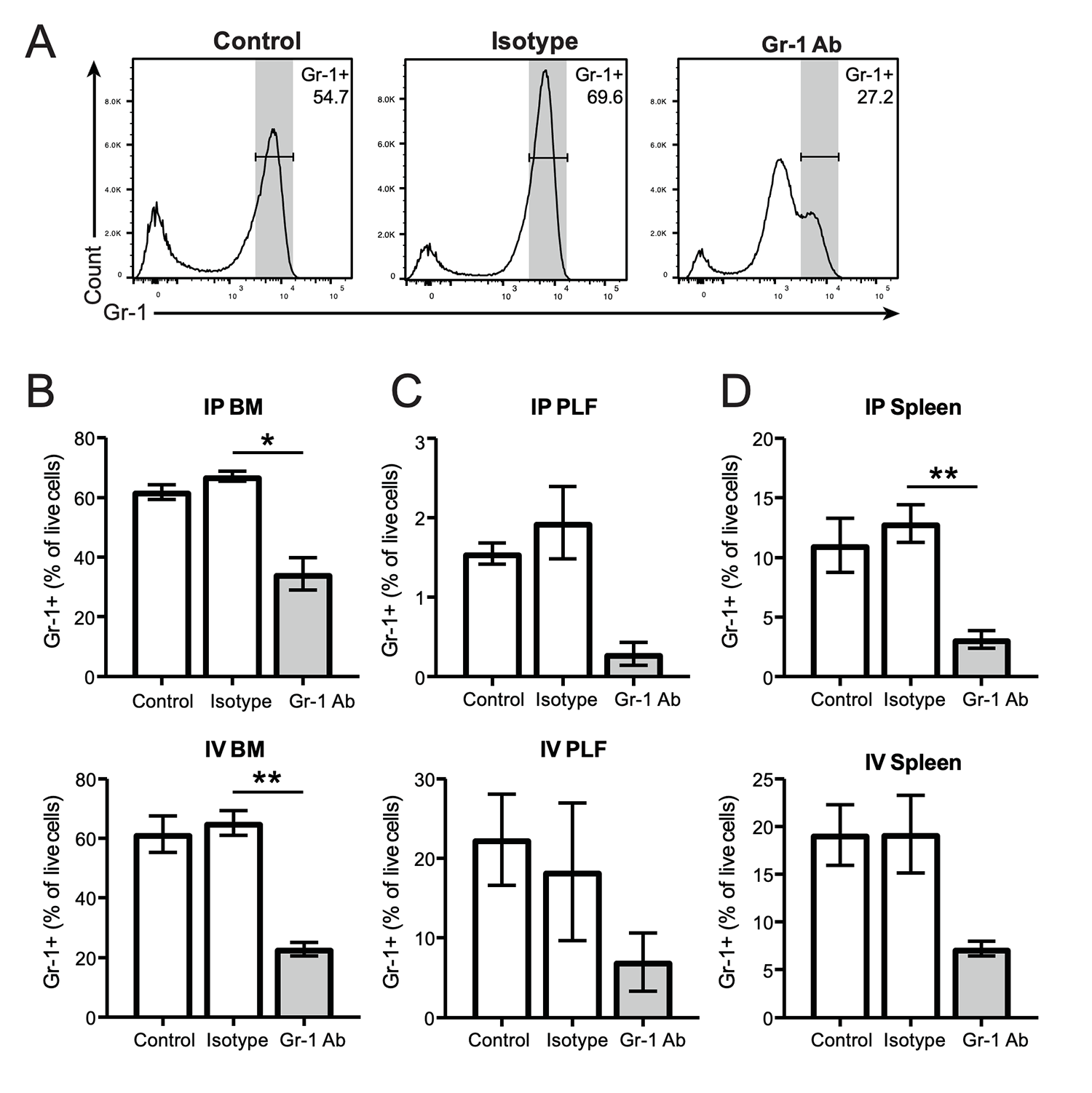

Supplement: FIG S2 [file mbio.02548-21-sf002.tif]

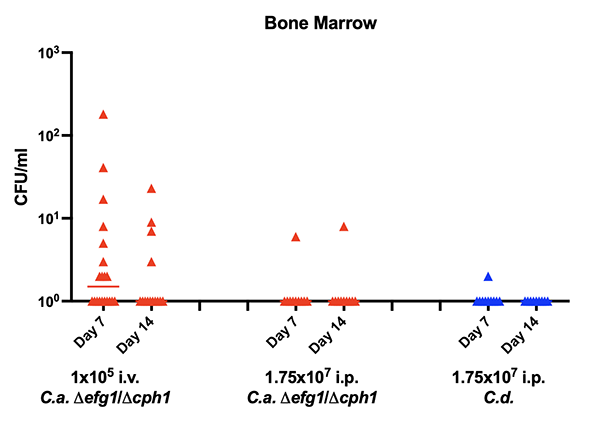

Supplement: FIG S3 [file mbio.02548-21-sf003.tif]
